# Effectiveness, safety and costs of orphan drugs: an evidence-based review

**DOI:** 10.1136/bmjopen-2014-007199

**Published:** 2015-06-24

**Authors:** Igho J Onakpoya, Elizabeth A Spencer, Matthew J Thompson, Carl J Heneghan

**Affiliations:** 1 Nuffield Department of Primary Care Health Sciences, University of Oxford, Centre for Evidence-Based Medicine, Oxford, UK; 2 Department of Family Medicine, University of Washington, Seattle, USA

**Keywords:** GENERAL MEDICINE (see Internal Medicine), PRIMARY CARE, ORPHAN DRUGS

## Abstract

**Introduction:**

Several orphan drugs have been approved by the European Medicines Agency (EMA) over the past two decades. However, the drugs are expensive, and in some instances, the evidence for effectiveness is not convincing at the time of regulatory approval. Our objective was to evaluate the clinical effectiveness of orphan drugs that have been granted marketing licenses in Europe, determine the annual costs of each drug, compare the costs of branded orphan drugs against their generic equivalents, and explore any relationships between orphan drug disease prevalence and annual costs.

**Methods:**

We searched the EMA database to identify orphan drugs granted marketing authorisation up to April 2014. Electronic searches were also conducted in PubMed, EMBASE and Google Scholar, to assess data on effectiveness, safety and annual costs. 2 reviewers independently evaluated the levels and quality of evidence, and extracted data.

**Results:**

We identified 74 orphan drugs, with 54 (73%) demonstrating moderate quality of evidence. 85% showed significant clinical effects, but serious adverse events were reported in 86.5%. Their annual costs were between £726 and £378 000. There was a significant inverse relationship between disease prevalence and annual costs (p=0.01); this was largely due to the influence of the ultra-orphan diseases. We could not determine whether the balance between effectiveness and safety influenced annual costs. For 10 drugs where generic alternatives were available, the branded drugs were 1.4 to 82 000 times more expensive.

**Conclusions:**

The available evidence suggests that there is inconsistency in the quality of evidence of approved orphan drugs, and there is no clear mechanism for determining their prices. In some cases, far cheaper generic agents appear to be available. A more robust, transparent and standard mechanism for determining annual costs is imperative.

**Strengths and limitations of this study**

- We employed a robust strategy to search for the best quality evidence for effectiveness.
- We used standardised methods to rate the quality and level of evidence for each orphan drug.
- We also used reliable data to document the prevalence of each orphan disease and identify the annual cost of each orphan drug.
- Because we could not document the research and development costs associated with the approval of each orphan drug, the influence of this variable on their annual costs could not be ascertained.
- The inconsistencies in the quality of evidence for some orphan drugs limit the conclusions that could be drawn regarding their effectiveness and safety.

## Introduction

Orphan drugs are therapeutic agents designed for the management of orphan diseases^1^ which are defined as medical conditions with a low prevalence. In the USA, orphan diseases are defined as those affecting approximately 1 in 1500 persons,^2^ while in Europe they are defined as those affecting 5 in 10 000.^2^ ^3^ In Europe, ultra-orphan designations are used to specify rare diseases with prevalence of less than 1 in 50 000.^2^ ^4^

Because low patient volumes make it unfavourable for commercial investment in orphan drug research, drug regulatory authorities have incentives in place to encourage their development and manufacture by pharmaceutical companies. In the USA, these include exclusive licensing to market such drugs for 7 years, faster assessment procedures, and tax incentives.^1^ In Europe, incentives include exclusive licensing for 10 years, reduction in the fees paid for regulatory activities, and provision of scientific advice by drug regulatory bodies.^1^ ^5^ Despite these incentives, the costs of orphan drugs are still high, especially when generic versions of the same agent are available.^6^ In the UK, for example, some funders of care under the National Health Service (NHS) have refused to fund orphan drugs because they are not considered cost-effective, thereby denying patients access to potentially useful drug interventions.^7^ Factors that reportedly influence their price setting include research and development (R & D) costs, clinical effectiveness, drug quality and disease prevalence.^8^ ^9^ However, the effectiveness of some orphan drugs has not been clearly demonstrated, and the evidence regarding their safety is often sparse at the time of regulatory approval.^10^

A previous systematic review of 11 orphan drugs approved in the Netherlands concluded that there is scarcity of information on the cost-effectiveness of the drugs.^11^ A more recent systematic review of orphan drug legislation in Europe also advocated for more stringent approval criteria for evaluating the clinical and cost-effectiveness of orphan drugs.^12^ Another systematic review of orphan drugs used in cancers showed that they have varying levels in quality of evidence and dearth of information on economic value.^13^ While the first two reviews did not evaluate the quality of the evidence, the third review focused only on drugs marketed in the USA.

The objective of this review was to evaluate the effectiveness and safety of all orphan drugs that have been granted marketing licenses in Europe, determine the annual costs of each drug based on UK estimates, compare the costs of branded orphan drugs against their generic equivalents, and explore the relationship between prevalence of orphan disease and annual costs.

## Methods

We searched the European Medicines Agency (EMA) database for orphan drugs and their approved indications using the search modality ‘http://www.ema.europa.eu/ema/>find medicine>human medicines>Browse by type>Orphan medicine: “Include authorised medicine, withdrawn post-approval and suspended” >SUBMIT’. Orphan drugs granted marketing authorisation were identified up to 30 April 2014. Drugs that have been designated ‘orphan status’, but have not received EMA marketing authorisation, were excluded. The EMA Orphanet Report Series (April 2014)^14^ was also assessed to verify approvals of the identified drug.

For each identified drug, electronic searches were then conducted in the following databases: PubMed, EMBASE, the Clinical Trials database and National Electronic Library for Medicines. The search terms used included: “orphan drug name” AND “meta-analysis”, systematic review”, “randomised”, “clinical trial”, “placebo”, “follow-up study”, “retrospective studies”, “case-series”, “cohort”, “follow-up studies”, “adverse events”. The prevalence of orphan diseases was documented using the reference values reported in the Orphanet Report Series Bibliographic data,^15^ and the Orphanet portal for rare diseases and orphan drugs.^16^

We estimated the annual average cost in the UK for each orphan drug. Costs of orphan drug regimens might vary according to the individual patient's needs including body size, disease progression, or complications of disease. However, we did not have information on these factors, and so we used the most recent data to estimate the average annual cost in the UK. We searched the UK Medicines Information, the National Electronic Library for Medicines, North East Treatment Advisory Group (NETAG), Scottish Medicines Consortium, and All Wales Medicines Strategy Group databases, for the most recent evidence relating to the annual costs of each drug. Where these were inadequate to compute the annual costs of a specific drug, we searched The Pharma Letter (http://www.pharmaletter.com) and PharmaTimes (http://www.pharmatimes.com) websites, and Google Scholar, for the most recent data on annual costs. The costs of treatment with drugs not used on an annual basis/duration were computed as annual costs. Where orphan drugs were approved for two or more indications, we documented the annual costs of treatment separately for each indication.

For each orphan drug identified from the EMA database, we determined the level of available evidence regarding effectiveness using the Oxford Centre for Evidence-Based Medicine (OCEBM) levels of Evidence,^17^ which comprises four levels: level 1: systematic reviews; level 2: randomised trial or observational study with dramatic effect; non-randomised controlled cohort/follow-up study; level 3: case series, case control studies, or historically controlled studies; level 4: mechanism-based reasoning.

The quality and strength of the overall body of evidence for each orphan drug was then evaluated using a checklist adapted from the Grades of Recommendation, Assessment, Development and Evaluation (GRADE) criteria,^18^ which assesses the five domains: study design; consistency of evidence; directness of the evidence; precision; and reporting biases. On the basis of the quality of overall evidence, one of four possible grades could be allocated: high, moderate, low and very low. Evidence for effectiveness for each orphan drug from the highest levels of evidence for each orphan drug was prioritised using the following order: meta-analysis/systematic review>RCTs>non-randomised studies. If two or more systematic reviews evaluated the same orphan drug, the most recent review was included. If two or more drugs were approved for treating the same orphan disease, we determined whether the level of effectiveness was related to their annual costs. Scatter plots were used to explore the relationships between prevalence of orphan disease and annual costs. If three or more drugs were approved for the same indication, we used scatter plots to test whether there was a relationship between year of approval and the annual cost. Where systematic reviews or meta-analyses did report adverse events, we searched for adverse event publications using the evidence from systematic searches using the following priority: RCTs>retrospective/follow-up/cohort>case-control/case-series. Adverse events (≥Grade 3) associated with the use of such drug were documented. For drugs that had been withdrawn or suspended, we documented the reasons for such decisions by accessing the EMA European Public Assessment Reports (EPAR) for human medicines.^19^

Two reviewers (IJO and EAS) independently evaluated the level, quality and strength of the evidence, and extracted data. These were then cross-checked by two other reviewers (MJT and CJH). Disagreements were resolved through consensus.

## Results

**Figure 1:**
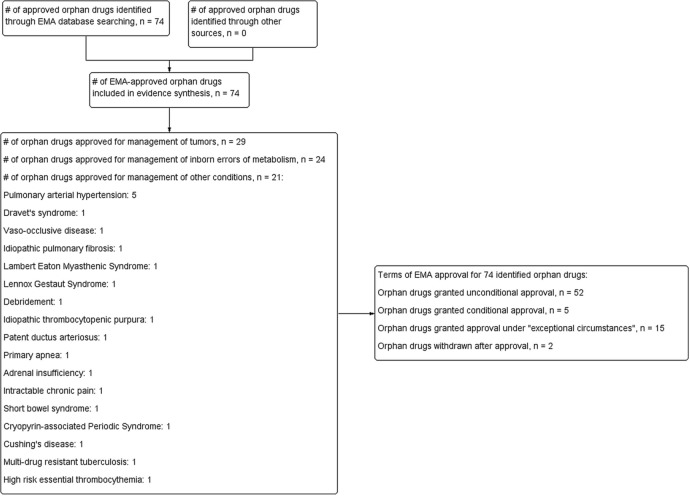
Flow chart showing process for inclusion of European Medicines Agency (EMA)-approved orphan drugs.

The EMA database searches identified a total of 74 approved drugs (authorised medicines or medicines withdrawn postapproval) for managing 63 orphan conditions, with approval dates ranging from 15 May 2002 to 04 April 2014 (figure 1). Twenty-nine (39%) of these drugs are used in the management of cancers, while 24 (32.4%) are used for inborn errors of metabolism or immune disorders. The remaining 21 drugs were approved for a variety of other orphan conditions, with pulmonary arterial hypertension accounting for 5 (23%) of the approvals. Of the 74 drugs, 5 (6.8%) were granted conditional approval, while 15 (20%) were granted approval under ‘exceptional’ circumstances. Two drugs, Cholic acid FGK and Orphacol, both contained the same active ingredient and were approved for the same indication; however, Orphacol was granted license under ‘exceptional’ circumstances. Two drugs, Onsenal and Rilonacept, were withdrawn by the EMA after approval because of an unfavourable risk-to-benefit profile, and commercial reasons, respectively. Details of the approval dates, levels and quality of evidence, results of clinical effectiveness, annual costs and relevant references have been included as web appendices 1–4.

### Levels and quality of evidence

**Figure 2:**
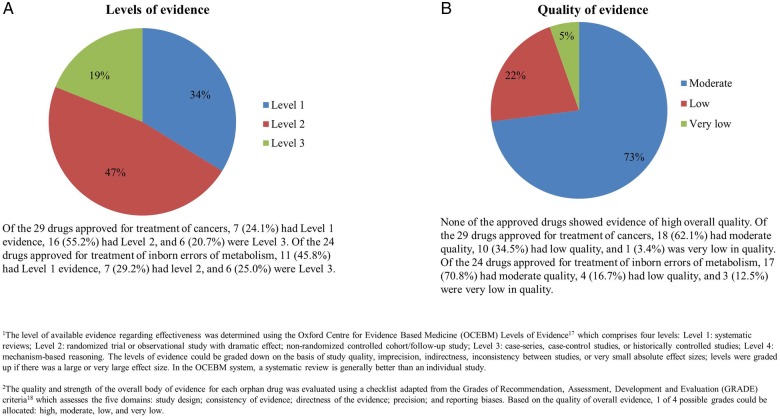
Pie charts showing the (A) levels and (B) quality of evidence of European Medicines Agency (EMA)-approved orphan drugs by proportion.

Using the OCEBM criteria, 25 (33.8%) orphan drugs had level 1 evidence, 35 (47.3%) were level 2, and 14 (18.9%) were level 3 (figure 2A). Basing on the GRADE criteria, the overall quality of evidence could be rated as moderate in 54 (73.0%) drugs, low in 16 (21.6%) and very low in 4 (5.4%) (figure 2B). None of the 74 drugs showed evidence of high overall quality. Relevant references for the level and quality of evidence are included as web appendix 1.

### Evidence for effectiveness

Of the 29 drugs approved for management of cancer, 6 (20.7%) showed evidence of significant benefits for both progression-free survival and overall survival; 7 (24.1%) showed evidence of significant improvement only in progression-free survival, while 9 (31.0%) had evidence of only significantly increasing overall survival (see web appendix 2 and table S1). Of the 24 orphan drugs approved for treating inborn errors of metabolism, 21 (75%) showed evidence of beneficial effect on at least one outcome measure (see web appendix 2 and table S2). Of the remaining 21 drugs used for managing other orphan diseases, 20 (95.2%) showed evidence of significant beneficial effects (see web appendix 2 and table S3).

### Evidence for safety

Twenty-eight (96.6%) of the orphan drugs approved for treating cancerous conditions had evidence of serious adverse events (see web appendix 2 and table S1). The most common events were bone marrow suppression (58.6%) and hepatotoxicity (20.7%). Adverse events with Dacogen and Evoltra were severe enough to warrant premature termination of clinical trials.

Six of 24 drugs (25%) approved for treating inborn errors of metabolism had no evidence of serious adverse events, while another (Cholic acid) did not have any preclinical safety studies prior to approval (see web appendix 2 and table S2). The most common events involved the gastrointestinal and respiratory systems (25% and 20.8%, respectively).

Eighteen of the 21 drugs (85.7%) approved for treating other orphan conditions had evidence of serious adverse events (see web appendix 2 and table S3). Gastrointestinal adverse events were the most common (23.8%). Other notable adverse events included cardiotoxicity (9.5%), metabolic abnormalities (9.5%) and possible risk of suicide with Prialt (ziconotide).

### Annual costs of orphan drugs

The annual cost ranged between £726 and £378 000 (median £31 012) (details of the annual costs and the references used for documenting the evidence are shown in web appendix 3). Twenty-four per cent of the drugs cost less than £10 000 annually, 58% cost between £10 000 and £100 000, while 18% cost ≥£100 000 annually. For cancer drugs, the range was £1800 to £92 000, compared with £726 to £378 000 for inborn errors of metabolism.

### Association between disease prevalence and annual cost of orphan drugs

**Figure 3:**
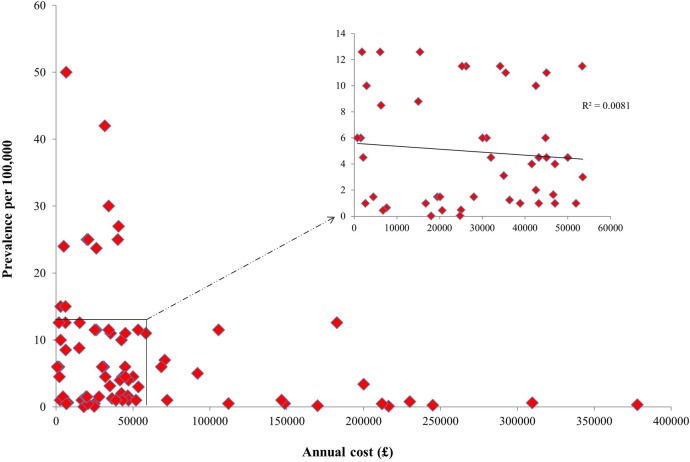
Scatter plot of orphan disease prevalence against annual cost.

**Figure 4:**
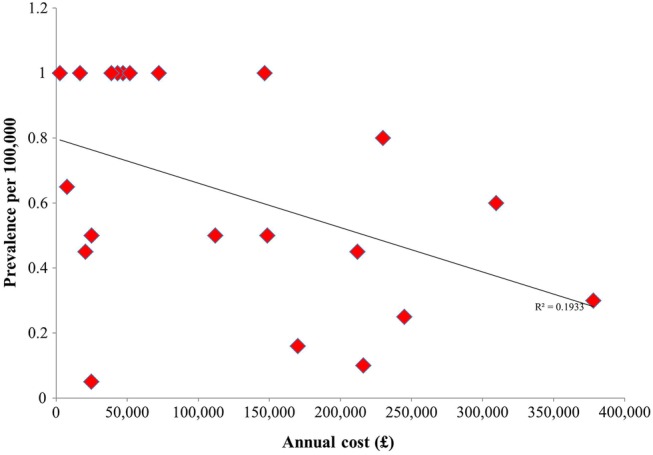
Scatter plot of ultra-orphan disease prevalence against annual cost.

A scatter plot of prevalence against annual cost (figure 3) revealed a significant inverse relationship (p=0.01). By contrast, we did not observe a significant relationship when a subarea of more frequent activity (similar range of disease prevalence and annual costs) was analysed (p=0.56; figure 3 inset). A significant inverse association was also observed for the relationship between annual cost and the prevalence for 21 drugs approved for managing ultra-orphan diseases (p=0.04; figure 4). However, a scatter plot of the subset of the 53 drugs approved for orphan diseases with prevalence >1 per 100 000 revealed a non-significant relationship (p=0.18). Scatter plots of prevalence against annual cost did not reveal significant relationships when cancers or inborn errors of metabolism were individually tested (data not shown).

### Association between clinical effectiveness and annual cost

Because of discrepancies in outcome measures and time points for outcome measurements, we could not use scatter plots to explore associations between clinical effectiveness and annual costs. All orphan drugs approved for managing pulmonary arterial hypertension showed comparative level of effectiveness at improving 6 min work distance, and decreasing clinical worsening irrespective of annual cost (see web appendix 4). Similar findings were observed for progression-free survival and overall survival for orphan drugs approved for treating cancers. The annual costs of two drugs approved for treating Pseudomonas in cystic fibrosis were comparable. We could not determine whether the risk or occurrence of serious adverse events played a role in the annual costs of approved orphan drugs.

### Association between year of approval and annual cost

Two or more orphan drugs were approved by the EMA for treating seven orphan conditions (see web appendix 4). Scatter plot of annual cost against year of approval for the five drugs approved for pulmonary arterial hypertension suggested a trend towards a significant relationship for higher annual costs with more recent approvals (p=0.06). A significant relationship between annual cost and year of approval was observed with four drugs approved for the management of chronic myeloid leukaemia (p=0.03). There was no significant relationship between annual cost and year of approval for drugs approved for treatment of acute lymphoblastic leukaemia (p=0.38); however, exclusion of the most recently approved drug of which approval was based on a historical perspective (Xaluprine) resulted in a significant relationship being observed (p=0.01).

### Branded orphan drugs versus generic or unlicensed versions

**Figure 5:**
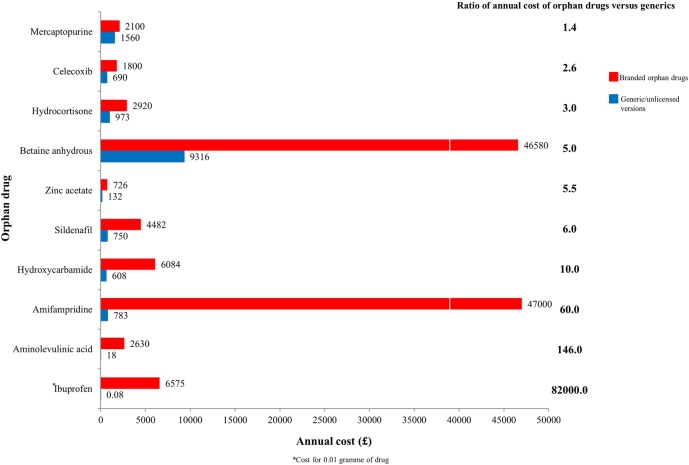
Price comparison of branded orphan drugs versus generic/unlicensed versions.

We found 15 approved drugs with generic versions, of which data on annual cost of generic or unlicensed versions for 10 (13.5%) were available. Figure 5 shows a price comparison in annual costs of branded orphan drugs compared with their unlicensed/generic equivalents. While branded mercaptopurine (Xaluprine) was only 1.4 times more costly than its generic counterpart, the branded version of intravenous ibuprofen (Pedea), used for closure of patent ductus arteriosus (PDA) in preterm infants, was 82 000 times more expensive than its oral equivalent.

## Discussion

### Main findings

Our results show that, of the 74 EMA-approved orphan drugs over a 12-year period, none has shown an overall high quality in available evidence for their effectiveness; there is a moderate level of evidence for three-quarters, while a fifth is low in evidence. Our analyses of annual drug costs revealed several interesting and potentially concerning findings. We found a significant inverse relationship between annual costs of orphan drugs and the prevalence of orphan diseases; in other words, the drugs for the most rare diseases are cheaper than those for more common diseases, largely driven by the drug costs for the ultra-orphan diseases. We also found that drugs approved more recently cost more than those approved over the previous decade. Finally, the annual costs of the drugs did not appear to be related to their clinical effectiveness, and for the subset of the branded orphan drugs that had generic equivalents, we found several extremely large differences in cost.

While the clinical trial evidence for over two-thirds of EMA-approved orphan drugs suggests that they have clinically significant beneficial effects, the evidence for about one-fifth indicates that either they do not have clinically beneficial effects, or, more importantly, they may do harm. The results of systematic reviews for several of the drugs are also inconclusive, either because of deficiency in trial reporting or due to a lack of available clinical trials. Our inability to determine whether the level of clinical effectiveness influences annual costs for orphan diseases with two or more approved indications indicates uncertainty about how this variable determines price setting.

The findings from our review show that except for the very rare (ultra-orphan) diseases, there is no significant inverse relationship between disease prevalence and annual cost. There were inconsistencies in the strengths of correlation between year of approval and annual costs when three or more drugs used for the same orphan condition were compared. This indicates that there is no standardised process for setting the prices of orphan drugs. It is important to realise that many orphan drugs target very small patient numbers, and are for conditions with few treatment alternatives, thereby allowing their prices to be relatively higher than non-orphan drugs.

### Comparison with the existing literature

The results of our review confirm a previous research report, which concluded that prevalence of orphan disease is inversely associated with annual costs for only very rare diseases.^8^ Our results also corroborate results from a previous review which showed that approved orphan drugs vary in the quality of evidence,^13^ and support the evidence from two previous reviews which suggested that there is no clarity about how clinical effectiveness is used to determine the costs of orphan drugs.^11^ ^12^ Contrasting with previous reports, our review is more systematic, and by far more comprehensive. We also determined whether year of approval has any influence in determining costs, and we compared branded versions of the orphan drugs with their unlicensed or generic versions. Our results support the findings of a qualitative analysis examining reimbursement for orphan drug prescriptions in Belgium, which suggested that more standardisation of how orphan drugs are priced is needed.^20^ However, we investigated all orphan drugs approved for use in Europe, and assessed the quality of evidence for effectiveness and safety.

### Strengths and limitations

We employed a robust strategy to search for the best quality evidence for effectiveness, safety and annual costs of each orphan drug, and we used standardised methods to rate the quality and level of evidence for each identified drug. We also used reliable data to document the prevalence of each orphan disease and identify the annual cost of each orphan drug.

However, we do recognise several limitations. Because we could not document the R&D costs associated with the approval of each orphan drug, the influence of this variable on their annual costs could not be ascertained. The heterogeneity in clinical outcomes across different diseases, discrepancies in time points for outcome measurements, and the inconsistency in the levels of outcome reporting, also limited the type of analyses and, therefore, the conclusions that could be drawn regarding the effectiveness and safety of some orphan drugs.

### Implications for research

Our finding that the trial evidence for the effectiveness and safety of 65% of drugs has not been systematically reviewed suggests an urgent need for prioritising reviews for these drugs. Moreover, systematic reviews evaluating the effectiveness and safety of some EMA-approved orphan drugs are now outdated because more recent clinical trials have become available, and, consequently, such reviews need to be updated. Given the value of systematic reviews for clinicians and health policymakers, we suggest the formation of a Cochrane Collaboration review group focused on orphan drugs as one way of reducing these gaps in knowledge.

In addition, further independent and industry-funded trials are also warranted, especially for drugs that currently have poor quality and low levels of evidence. Clinical trial investigators should adequately report any suspected adverse events observed in the course of trials; and statements made in some primary studies that such events, if any, were not related to the drug in question (or not serious enough) may be premature. The importance of close postapproval monitoring cannot be overemphasised. Finally, given the inconsistencies we found in drug costs, a more detailed and transparent analysis of the relationship between R&D costs, and annual costs of orphan drugs, is now imperative.

### Implications for policy

The use of novel therapeutic agents has resulted in improvements in clinical outcome for several orphan diseases. It is also rational that drug companies should aim to recoup investment in R&D costs through sales and reimbursements for these drugs, even though the size of the market for such drugs is quite small.^21^ However, the overall benefits on clinical and economic terms need to be taken into consideration when setting prices.^22^ In fact, unfavourable cost-to-benefit analysis has led to the rejection of applications for approval of orphan drugs by the UK National Institute for Health and Care Excellence.^23^ ^24^ Recently, NHS England threatened to stop purchasing some orphan drugs used for cancer management, unless the manufacturers reduced their prices.^25^ Since the cost-effectiveness of orphan drugs is difficult to assess, considering opportunity costs might help in making decisions about funding the provision of orphan drugs.

The high costs of orphan drugs are reportedly due to the large amounts of funds invested in R&D by the drug manufacturers.^26^ However, Light and Lexchin^27^ reported that drug companies spend only 1.3% of their revenue on basic research in order to discover novel molecules. In addition, the disparity in prices when these branded drugs are compared with their generic versions makes it doubtful if the quoted R&D costs are sufficiently robust. It is unclear in respect of the extent to which the incentives offered by the EMA and other drug regulatory authorities to encourage the development of these drugs influenced the prices of these branded drugs. For example, Pedea costs 82 000 times as much as generic ibuprofen, yet, research evidence has shown that Pedea is not superior to oral ibuprofen in clinical effectiveness regarding closure of PDA.^28^ This raises the question of whether large national health systems, such as the NHS, should choose generic versions of orphan drugs where available, particularly given current pressure on healthcare expenditures.^29^ In 2013, for example, the drug company Novartis AG lost its patenting right for Glivec (imatinib) in India, largely due to its very high price compared to its generic alternative.^30^

The marketing authorisations granted to some orphan drugs, such as Ceplene, Firdapse and Xaluprine, were based on a historical perspective, that is, these compounds have previously been used in patient management prior to being designated orphan status.^31^ Therefore, in cases such as these, it does not seem plausible for drug companies to claim huge R&D costs because they are not new therapies. Indeed, the application for approval of Ceplene for its designation as an orphan drug was refused in Canada due to a failure of the drug company to prove that its development was innovative.^32^ Similarly, it would be expected that the prices of drugs for which there are alternative therapeutic options for their stated indications would be competitively based.

The postapproval withdrawal of some orphan drugs suggests that premarketing and postmarketing surveillance is not stringently assessed (or evaluated) during the process of clinical trial and drug approval. For example, Onsenal (celecoxib) was also withdrawn 8 years postapproval after it was discovered that the risks outweighed its benefits in its use for managing familial adenomatous polyposis.^33^ Thelin (sitaxentan) lost its orphan status (and was withdrawn from the market) due to fatal hepatotoxicity;^34^ clinical trial results had shown that the drug had adverse effects on the liver, but these were not considered serious enough to prevent its approval. The serious adverse events associated with some approved drugs lend credence to the view that safety does not appear to be a factor when determining the costs of the drugs.

## Conclusion

There is inconsistency in the level and quality of evidence for approved orphan drugs. While some orphan drugs have demonstrated evidence of significant benefits, evidence of effectiveness is lacking for several others, and some are associated with serious unwanted adverse effects. The available evidence suggests that, except for the ultra-orphan diseases, the annual costs of orphan drugs approved in Europe are not influenced by disease prevalence. There is inadequate data to determine whether clinical effectiveness influences the price setting of orphan drugs. Further research into the effectiveness and safety of orphan drugs is required, and a standard, transparent and robust mechanism for determining their prices should be a priority.
